# Adeno-associated vector corneal gene therapy reverses corneal clouding in a feline model of mucopolysaccharidosis VI

**DOI:** 10.1371/journal.pone.0338370

**Published:** 2025-12-05

**Authors:** Brian C. Gilger, Liujiang Song, Tomoko Hasegawa, Jacklyn H. Salmon, Jacquelyn J. Bower, Naveen Vridhachalam, Elisabeth Collins, Darby Roberts, Jessica Bagel, Caitlyn Molony, Charles Vite, Matthew L. Hirsch

**Affiliations:** 1 Clinical Sciences, North Carolina State University, Raleigh, North Carolina, United States of America; 2 Department of Ophthalmology, University of North Carolina at Chapel Hill, North Carolina, United States of America; 3 Carolina Eye Research Institute, University of North Carolina at Chapel Hill, North Carolina, United States of America; 4 Center for Molecular Medicine, University of North Carolina at Chapel Hill, North Carolina, United States of America; 5 Lineberger Comprehensive Cancer Center, University of North Carolina at Chapel Hill, North Carolina, United States of America; 6 Department of Referral Center for Animals Models, University of Pennsylvania, Philadelphia, Pennsylvania, United States of America; 7 Department of Pathobiology, University of Pennsylvania, Philadelphia, Pennsylvania, United States of America; 8 Department of Small Animal Clinical Sciences, College of Veterinary Medicine, University of Florida, Gainesville, Florida, United States of America; Cedars-Sinai Medical Center, UNITED STATES OF AMERICA

## Abstract

Mucopolysaccharidosis VI (MPS VI) is a rare, autosomal recessive lysosomal storage disease caused by mutations in the arylsulfatase B gene (*ARSB*). Ocular manifestations of MPS VI include progressive corneal clouding, leading to vision loss. Herein, an adeno-associated virus (AAV) *ARSB* corneal gene addition strategy was evaluated in a naturally occurring MPS VI feline model. The AAV serotype 8 capsid was packaged with a single-strand optimized human *ARSB* expression cassette (opt*ARSB*) and administered to MPS VI feline corneas at a dose of 1e^9^ vector genomes via intrastromal injection. All AAV8-opt*ARSB* injections were well tolerated, resulting in a complete reversal of pre-existing corneal clouding within 2–3 weeks, which was maintained throughout the study. Sequential dosing of the contralateral cornea 7 weeks after the first dose also cleared the storage disease with similar kinetics despite more advanced disease. Confocal microscopy, histological analyses, and electron microscopy revealed disorganization in the posterior corneal stroma in untreated animals with AAV8-opt*ARSB*-treated corneas demonstrating improved morphology and tissue organization. Human arylsulfatase B was observed throughout the corneal stroma with decreased smooth muscle actin staining following AAV8-opt*ARSB* treatment. The collective results demonstrate that reversing feline MPS VI corneal clouding using intrastromal low-dose AAV8-opt*ARSB* is safe and effective. Furthermore, as this strategy relied on the same AAV capsid, vector dose, genetic cassette context, injection type, and volume deemed safe and effective for the treatment of MPS I, the data derived herein support a standardized pipeline for AAV corneal gene therapy.

## Introduction

Mucopolysaccharidosis type VI (MPS VI), or Maroteaux-Lamy syndrome, is a rare, autosomal recessive lysosomal storage disorder caused by a deficiency in the enzyme arylsulfatase B (*ARSB*). This enzymatic defect leads to the systemic accumulation of dermatan sulfate, a glycosaminoglycan (GAG), resulting in progressive systemic manifestations, including significant ocular involvement. Among the most visually debilitating features of MPS VI is corneal clouding, which arises from GAG deposition in the corneal stroma, leading to progressive opacification and vision loss typically beginning in early childhood [1].

Current therapeutic strategies for MPS VI include enzyme replacement therapy (ERT) with recombinant human ARSB (galsulfase) and hematopoietic stem cell transplantation (HSCT) [2]. While these interventions have demonstrated efficacy in ameliorating systemic symptoms of MPS VI, their impact on ocular manifestations remains limited due to the avascular nature of the cornea [3,4]. In advanced cases, corneal transplantation (penetrating or lamellar keratoplasty) may be employed to restore visual acuity, though long-term outcomes are often compromised by high rates of graft rejection in this patient population [5].

To address these limitations, several emerging therapies are currently under preclinical investigation for the treatment of corneal clouding associated with MPS diseases, including oral substrate reduction therapy and gene replacement using viral vectors [6,7]. In particular, a single-dose adeno-associated virus (AAV) IDUA gene replacement strategy administered to post-symptomatic MPS I canine corneal stroma reversed corneal opacity, an effect that was maintained throughout the study duration [7]. Additional studies in human corneas *ex vivo* and in wild-type rabbits further demonstrate the ability of AAV8 to mediate efficient human corneal gene delivery, highlighting the safety and restricted biodistribution of corneal gene therapy in general [8–11]. These findings underscore the potential of gene therapy to overcome the limitations of systemic treatments and offer durable correction of ocular pathology in MPS disorders.

To investigate if the successful AAV corneal gene addition strategy observed in MPS I canines could be extrapolated to reverse MPS VI corneal clouding, a naturally occurring MPS VI feline model was employed [12–14]. The ocular abnormalities of the MPS VI felines have been well defined and mimic human ocular physiology, corneal size, and MPS VI pathology [12–14]. Therefore, this study evaluated the tolerability and effectiveness of intrastromal injection of AAV serotype 8 (AAV8) encoding a human codon-optimized *ARSB* cDNA (opt*ARSB*) to resolve corneal clouding following a single intrastromal injection. Additionally, a sequential administration to the contralateral cornea 7 weeks after the initial injection was also investigated in an attempt to mimic early human clinical trial protocols. Using the same capsid, injection volume, and genetic regulatory elements as the MPS I canine experiments, with the sole difference being the encoded enzyme, corneal clouding was resolved in MPS VI felines within 3 weeks following a single injection, regardless of whether the subject was pre-dosed with the identical vector [7]. The clinical and postmortem analyses provide additional evidence for MPS VI storage disease resolution with improved corneal cell morphology and organization. The collective efficacy, vector biodistribution, and the ability for sequential dosing in MPS I canines and MPS VI felines using conserved AAV vector elements highlight a potential pipeline to expedite AAV therapeutics for treating corneal disease in general [7–9].

## Results

### opt*ARSB* overexpression confers arylsulfatase B activity that is well tolerated in human cells

**Fig 1:**
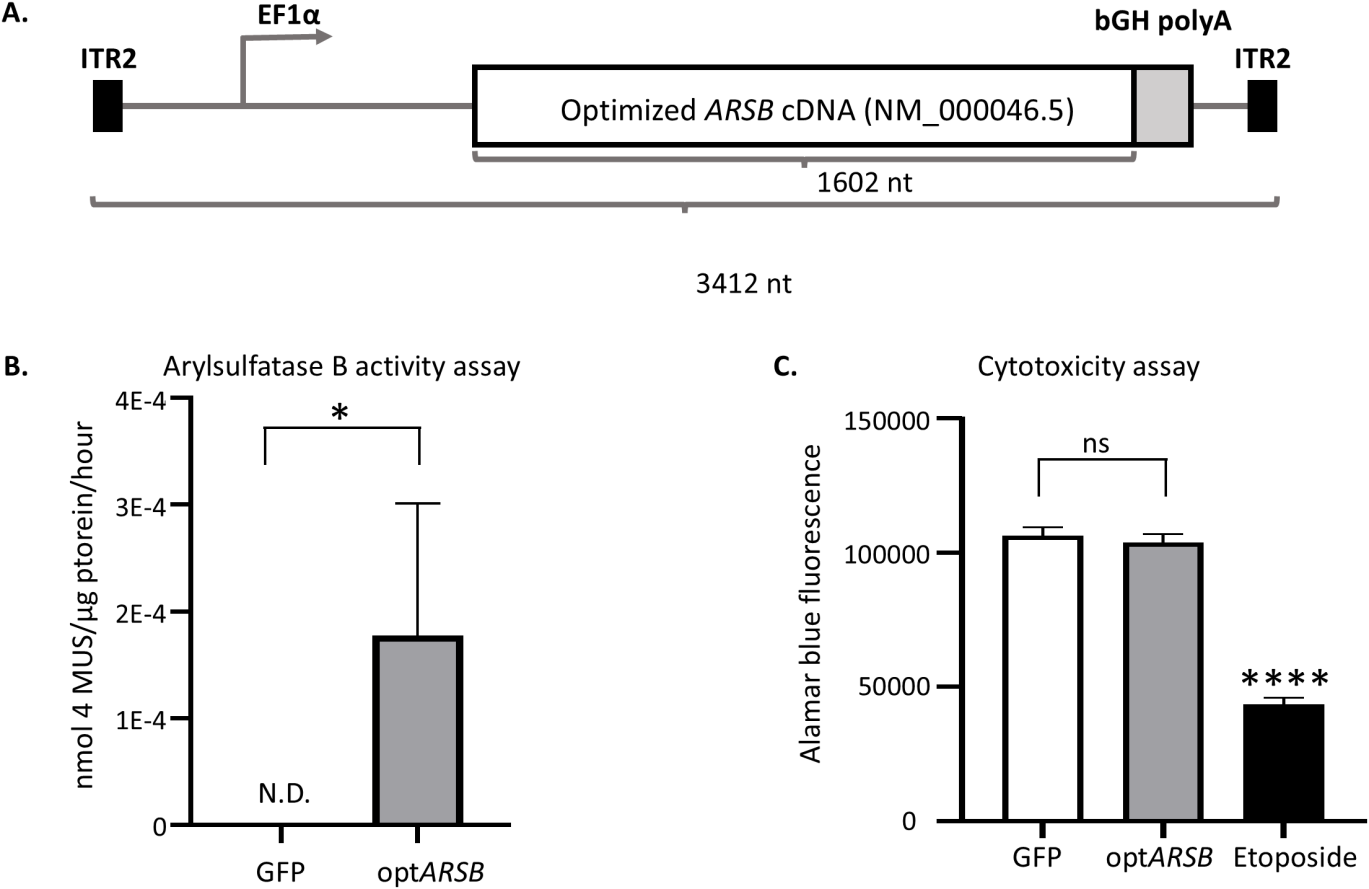
Construct Design. (A) A single-strand adeno-associated virus (AAV) genetic cassette was engineered by cloning a codon-optimized human ARSB (NM_000046.5) cDNA (opt *ARSB*) into a genetic cassette containing the ubiquitous EF1α human promoter and the bovine growth hormone polyadenylation [bGH poly(A)] sequence flanked by wild-type AAV2 inverted terminal repeats (ITR). (B) Arylsulfatase B activity was assessed using a colorimetric enzymatic activity assay. Protein harvested from HEK293 cells transfected with opt*ARSB* plasmid or GFP plasmid (control) was incubated with substrate, and the fluorescence intensity was measured and expressed as “nmol of 4-methylumbeliferyl sulfate (MUS)/µg of total protein/hour”. Graph is shown as mean with standard deviation. *p < 0.05, Student’s t-test. N.D., not detected. (C) An alamarBlue viability assay assessed cytotoxicity. HEK293 cells were transfected with the opt*ARSB* plasmid or GFP plasmid (control). Seventy-two hours after transfection, alamarBlue solution was added, and fluorescence was measured. A concentration of 20 µM etoposide was used as a cytotoxicity positive control. Graph is shown as mean with standard deviation. ns, no significant difference, Tukey’s HSD. ****p < 0.0001, GFP vs Etoposide and opt*ARSB* vs Etoposide, Tukey’s HSD.

Previous work demonstrated that AAV corneal gene therapy in an MPS I canine model reversed corneal opacity within 3–4 weeks following a single intrastromal injection; an effect that was maintained until the humane study endpoint [7]. Given these data and towards improving MPS VI patient vision, it was hypothesized that a similar approach fixing the variables of vector dose, genome context (promoter, poly(A), and single-stranded transgene), capsid, and administered volume, while substituting *IDUA* with *ARSB* cDNA, would reverse corneal clouding in an MPS VI model. Therefore, codon-optimized human *ARSB* (NM_000046.5) cDNA (opt*ARSB*) was cloned into a genetic cassette containing the ubiquitous EF1α human promoter and the bovine growth hormone polyadenylation sequence flanked by AAV serotype 2 inverted terminal repeats (Fig 1A). To verify arylsulfatase B function of this cassette, transfection experiments in HEK293 cells were reliant on a well-established colorimetric enzymatic arylsulfatase B activity assay [15]. The opt*ARSB* construct showed a significant amount of arylsulfatase B activity upon transfection compared to the control plasmid expressing the GFP reporter (Fig 1B). To determine if opt*ARSB* overexpression affected cell viability (or metabolism), an AlamarBlue cytotoxicity assay was performed. As expected, a significant decrease in AlamarBlue fluorescence was observed in etoposide-treated HEK293 cells while no significant difference was observed in cells treated with the GFP control or opt*ARSB* constructs (Fig 1C, S1 Fig).

### Characterization of the MPS VI Feline Ocular Phenotype

To test the therapeutic efficacy of AAV8-opt*ARSB*, a naturally occurring MPS VI feline model with an autosomal recessive, single missense mutation (L476P) in *ARSB,* resulting in non-functional arylsulfatase B, was employed [12]. This model develops progressive systemic lysosomal storage disease symptoms like those of patients with MPS VI, including progressive corneal clouding. Three separate heterozygous breeding attempts resulted in 10 offspring, 2 of which were homozygous for the *ARSB* L476P mutation and demonstrated an MPS VI phenotype (S1 Table). These affected felines, along with two asymptomatic heterozygous littermates, were transferred from the University of Pennsylvania to North Carolina State University at 75 days of age and used in the following experiments (Table 1).

**Table 1:** Animals used in this study.

Subject number	Sex	Genotype	Phenotype	Eye	Dose at Day 152	Dose at Day 206
Subject #1	F	*ARSB* ^-/-^	affected	OS OD	– AAV8-opt*ARSB* (1e^9^ vg)	– –
Subject #2	M	*ARSB* ^-/-^	affected	OS OD	AAV8-opt*ARSB* (1e^9^ vg) Saline	– AAV8-opt*ARSB* (1e^9^ vg)
Subject #3	F	*ARSB* ^ + /-^	non-affected	OS OD	Saline AAV8-opt*ARSB* (1e^9^ vg)	– –
Subject #4	M	*ARSB* ^ + /-^	non-affected	OS OD	– AAV8-opt*ARSB* (1e^9^ vg)	– –

Two homozygote (*ARSB*^-/-^, affected) and two heterozygote (*ARSB*^+/-^, non-affected) felines were dosed with Saline or AAV8-opt*ARSB* (1e^9^ vg) at 152 days of age. The right eye (OD) of Subject #2 was dosed with Saline at 152 days of age, followed by dosing with AAV8-opt*ARSB* (1e^9^vg) at 206 days of age (sequential dosing). OS: left eye, OD: right eye.

**Fig 2:**
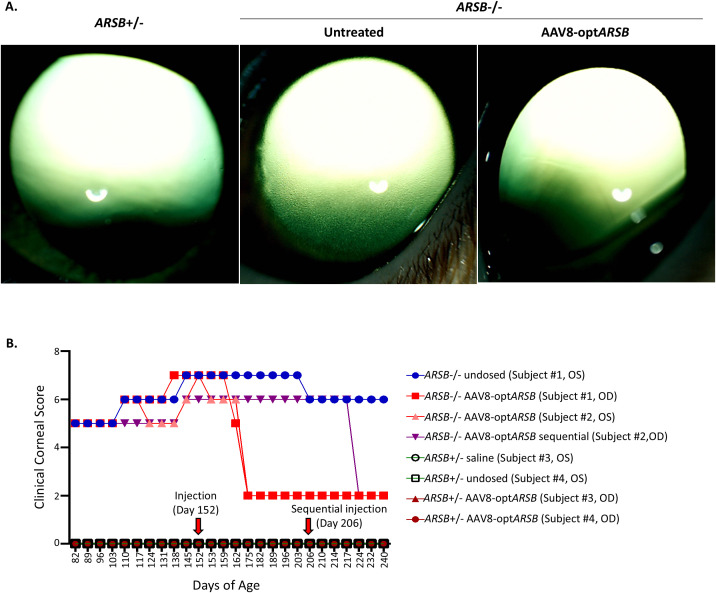
Corneal clearing after AAV8-optARSB in MPS VI felines. (A) Corneas from felines homozygous for the L476P mutation in the *ARSB* gene (*ARSB*^-/-^, affected MPS VI felines) were imaged 28 days after AAV8-opt*ARSB* intrastromal injection. Heterozygous feline cornea (*ARSB*^+/-,^ not affected control felines) is shown for comparison. (B) Clinical corneal scores, consisting of extent and area of corneal opacity and corneal vascularization, were recorded over the experiment period (from 82 to 240 days of age). The individual cornea score for each eye is shown. Corneas were dosed with intrastromal AAV8-opt*ARSB* (1e^9^ vg) or saline injection at 152 days of age. One homozygous eye (Subject #2, OD) was dosed with AAV8-opt*ARSB* at 206 days of age following a preceding AAV8-opt*ARSB* injection on the contralateral eye (Subject #2, OS) at 152 days of age (sequential dosing). OD: right eye, OS: left eye.

After acclimation, complete ophthalmic examinations, including ultrasonic pachymetry, corneal optical coherence tomography (OCT), and photography, were performed every week. Upon initial examination (82 days of age), homozygous felines had bilateral, mild, diffuse, corneal granular opacity (Fig 2). Slit lamp examination revealed that the corneal opacities were visible diffusely throughout the corneal stroma, with increased density in the deeper corneal lamellae. In contrast, the corneal epithelium and endothelium appeared normal. Although the homozygous felines did not demonstrate discomfort (e.g., blepharospasm or epiphora), they appeared photophobic compared to the heterozygous felines.

### Intracorneal administration of AAV8-opt*ARSB* is well tolerated in MPS VI felines

At 152 days of age, when subjects were large enough to undergo anesthesia (i.e., reduced chance of complications), the felines received intrastromal injections using a 31-gauge needle introduced tangentially into the cornea to deliver 50 µL of AAV8-opt*ARSB* (1e9 vg) or saline to the central corneal stroma [7,9]. No anterior chamber penetration or other injection-related complications were observed. The injections and subsequent clinical examinations were performed by a veterinary ophthalmologist who was masked to the identity of the test article administered. Immediately upon injection, the cornea became opaque in approximately 50% of the central cornea due to swelling of the stroma from the test article fluid. The injection-induced corneal opacity cleared within 2 hours post-injection. By 24 hours, the cornea returned to the pre-injection state of appearance, regardless of the test article administered (AAV8-opt*ARSB* or saline). Two heterozygote and two homozygote felines received a single unilateral intrastromal injection of 50 μL of rAAV8-opt*ARSB* (1e^9^ vg) in one eye, while the opposite eye was injected with saline or remained initially untreated at 152 days of age (Table 1). A homozygote male (Subject #2) received an additional injection of AAV8-opt*ARSB* (1e^9^ vg) in the eye at 206 days of age; the same eye had previously been dosed with saline, and the contralateral eye had been dosed with AAV8-opt*ARSB* (1e^9^ vg) at 152 days of age (sequential dosing) (Table 1). None of the felines showed signs of inflammation (i.e. redness or chemosis of the conjunctiva, discharge, aqueous cells or flare) throughout the study period other than one heterozygote female (Subject #3, left eye), dosed with saline, showed transient redness and chemosis of the conjunctiva 1 day after the injection. All felines had normal ocular funduscopic examination; however, the homozygous felines fundi were not clearly visible in untreated eyes, especially later in the study (after approximately 130 days of age). Intraocular pressure, as measured by tonometry, was normal in all felines throughout the study, consistent with our previous findings in MPS I canines [7].

### Intracorneal administration of AAV8-opt*ARSB* results in corneal clearing within 3 weeks

The homozygote corneas showed diffuse corneal opacity, which increased in density with age. However, the homozygote corneas treated with AAV8-opt*ARSB* became transparent and clear within three weeks post-injection, while the saline-injected or untreated cornea remained opaque (Fig 2A, S2 Fig A). Moreover, the clearing in homozygote corneas treated with AAV8-opt*ARSB* was limited to the area where the vector solution was actually injected, leaving the non-treated peripheral area opaque (S2 Fig C). The clinical corneal scores were comprised of measurements of the extent and area of corneal opacity and corneal vascularization, initially reflected the mild diffuse corneal opacity observed in homozygous felines that increased in density with age (Fig 2B). No heterozygous felines displayed visible ophthalmic abnormalities (including corneal opacity and inflammation) regardless of whether they were dosed with saline or AAV8-opt*ARSB* for the duration of the study period, other than the immediate post-injection ocular changes described above (Fig 2, S2 Fig A).

### Sequential intracorneal administration of AAV8-opt*ARSB* following contralateral dosing results in corneal clearing within 3 weeks

The cornea of the homozygous MPS VI feline that did not receive treatment demonstrated progressive corneal opacification (Fig 2, S2 Fig, Subject #1 OS, Table 1). Similarly, the cornea of the homozygous male who received a saline injection at day 152 demonstrated progressive corneal opacification (Fig 2, S2 Fig, Table 1, Subject #2 OD). To determine the therapeutic effect of AAV8-opt*ARSB* at a later stage of corneal disease progression, the homozygous feline that had previously been administered AAV8-opt*ARSB* in one cornea (Table 1, Subject #2 OS) was dosed with AAV8-opt*ARSB* in the contralateral cornea (Subject #2 OD, 1e^9^vg in 50μl; sequential dosing scheme) at day 206. Similar to the other AAV vector-treated corneas, this sequentially dosed affected cornea exhibited complete central corneal clearing within 3 weeks (S2 Fig B), despite further disease progression. Collectively, these data suggest that a single injection of AAV8-opt*ARSB* resulted in corneal clearing within three weeks, which was maintained throughout the remainder of the study (12 weeks). Additionally, at an interval of more than seven weeks following the first injection, a contralateral injection to a cornea at a later stage of disease progression resulted in the clearing of pre-existing corneal opacity.

### Corneal thickness assessment of AAV8-opt*ARSB* in MPS VI felines

Central corneal thickness (CCT), measured by an ultrasonic pachymeter, increased with age in the unaffected heterozygous subjects with values within the reference range for wild-type felines (S3 Fig A) [16]. In contrast, homozygous felines exhibited a CCT that was approximately 50–100 µm less in thickness than heterozygous subjects and did not increase in thickness with age through day 152 before AAV8-opt*ARSB* dosing (S3 Fig A). The CCT in homozygous eyes dosed with AAV8-opt*ARSB* demonstrated a trend of increasing CCT; however, they failed to achieve the corneal thickness values of the corneas of unaffected heterozygotes (S3 Fig A). Corneal OCT imaging from 99–204 days of age also showed the thinner CCT in homozygote corneas (P < 0.01), which was consistent with the pachymetry measurement (S3 Fig B). No differences in epithelial thickness were observed at any time point (S3 Fig B).

### Morphological corneal assessment of AAV8-opt*ARSB* in MPS VI felines by confocal microscopy

**Fig 3:**
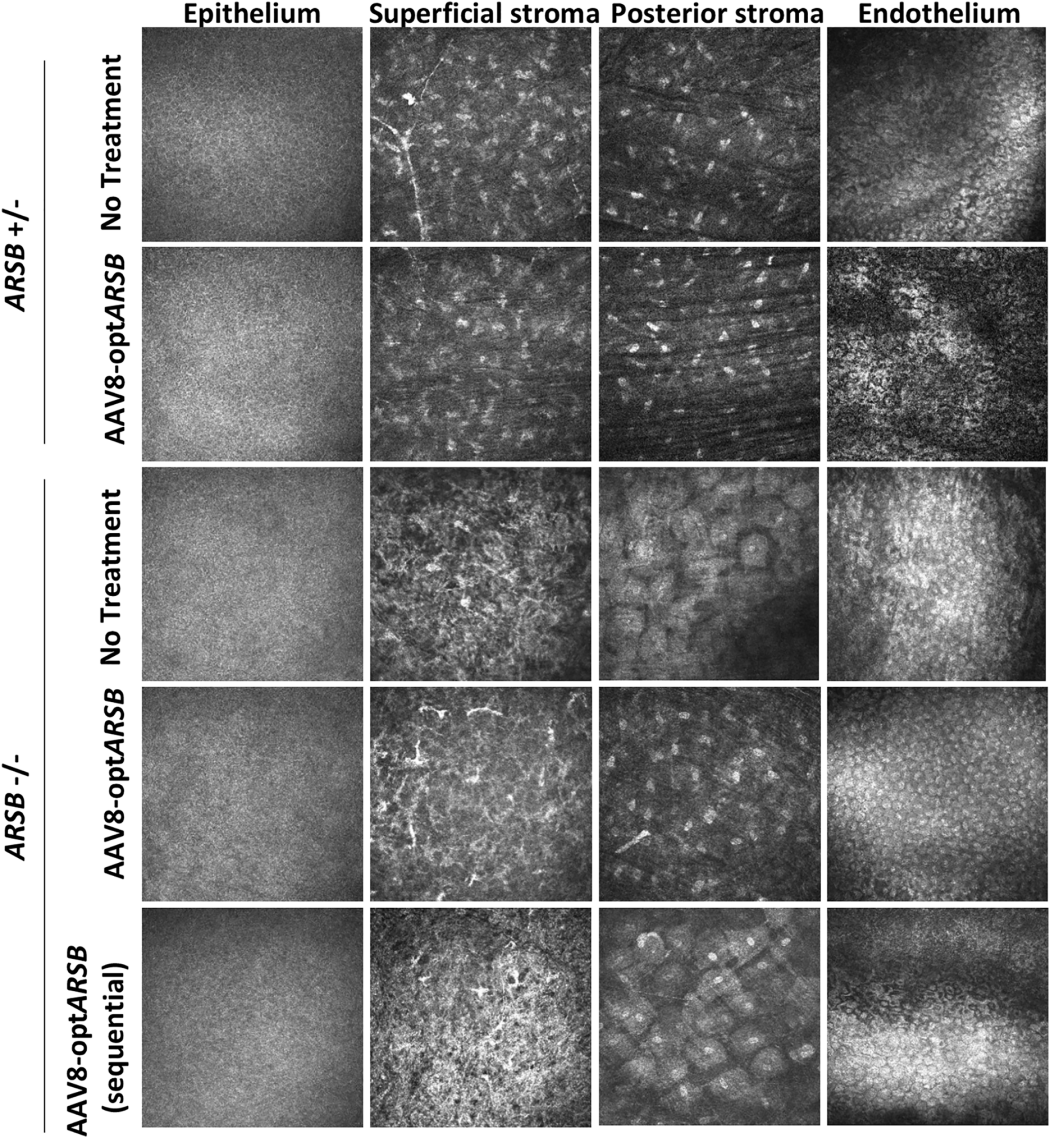
Confocal microscopy images of corneas in MPS VI felines. Epithelium layer, superficial stroma, posterior stroma, and endothelium in heterozygote (non-affected) felines and in homozygote (affected MPS VI) felines with or without AAV8-opt*ARSB* treatment were imaged at 242 days of age. The sequentially dosed eye, which was injected with AAV8-optARSB on day 206 of age, was also imaged on day 242 of age. Images from untreated and sequentially dosed homozygote posterior stroma show diffuse plump cells with increased cytoplasmic volume.

At 242 days of age, in vivo corneal confocal microscopy (CCM) imaging was performed. In the MPS VI heterozygote corneas, the superficial stromal area, which is located posterior to the basal epithelial nerve plexus, showed regularly arranged hyper-reflective cell nuclei, and stromal nerves were observed, similar to the previously reported images from healthy corneas (Fig 3) [17]. In the homozygote superficial corneal stroma, cells are more irregularly arranged (Fig 3). Treatment with AAV8-opt*ARSB* did not appear to alter this disorganization. CCM imaging of the untreated homozygote posterior corneal stroma revealed diffuse plump ovoid cells with increased cytoplasmic volume in the posterior corneal stroma with reduced cell numbers when compared to heterozygote images (Fig 3). In contrast, CCM imaging of the AAV8-opt*ARSB* treated homozygote posterior corneal stroma (injection at day 152, Subject #2) revealed cells with less or no intracellular material and improved overall organization similar to unaffected heterozygotes (Fig 3). The posterior corneal stroma of homozygous feline treated sequentially with AAV8-opt*ARSB*, imaged 36 days after the dosing (sequential injection day 206; imaging day 242), showed intracellular material storage similar to the untreated homozygote cornea; suggesting that it may take longer than 36 days after injection to clear the deep stroma cell layer of intracellular material (Fig 3). No difference was noted in heterozygote feline posterior corneal stroma, regardless of whether they were untreated or injected with AAV8-opt*ARSB*. Corneal endothelial cells counted by confocal microscopy did not decrease after AAV8-opt*ARSB* intrastromal injection (S2 Table).

### AAV8-opt*ARSB* treatment decreases GAG accumulation, evokes no inflammation, and improves fibril organization in MPS VI affected corneas

**Fig 4:**
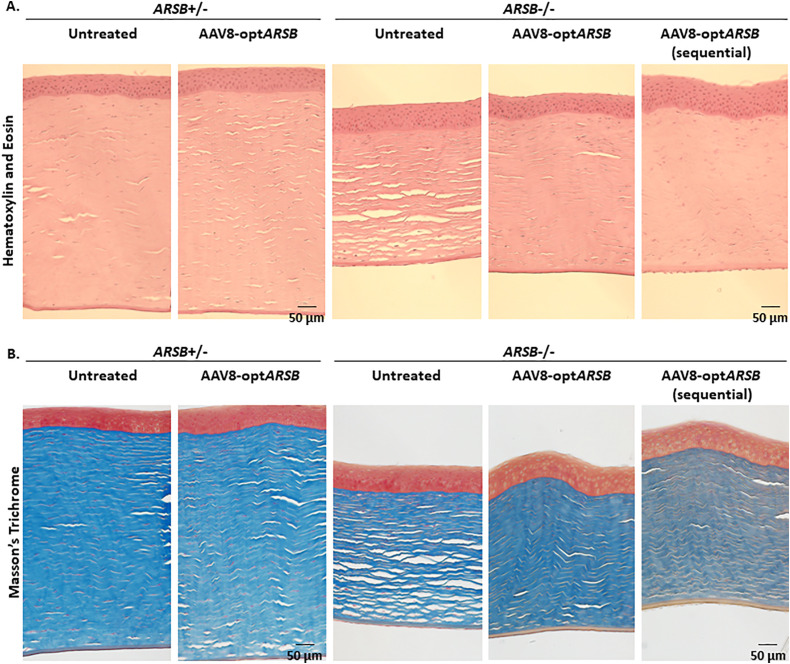
Histological evaluation of corneas in MPS VI felines. Hematoxylin and eosin (**A**) and Masson’s Trichrome (**B**) stained sections from heterozygote (non-affected, *ARSB*^+/-^) and homozygote (affected MPS VI, *ARSB*^-/-^) felines with or without AAV8-opt*ARSB* intrastromal injection are imaged. Scale bars: 50 µm.

Histological evaluations of the cornea showed no corneal abnormalities in the heterozygote felines (Fig 4, S3 Table)*.* In the untreated homozygote feline cornea, CCT was decreased with greater disorganization of the posterior stroma compared to the heterozygous tissue (Fig 4). No cellular infiltration or neovascularization was observed in homozygote or heterozygote corneas regardless of AAV8-opt*ARSB* injection (Fig 4, S3 Table).

**Fig 5:**
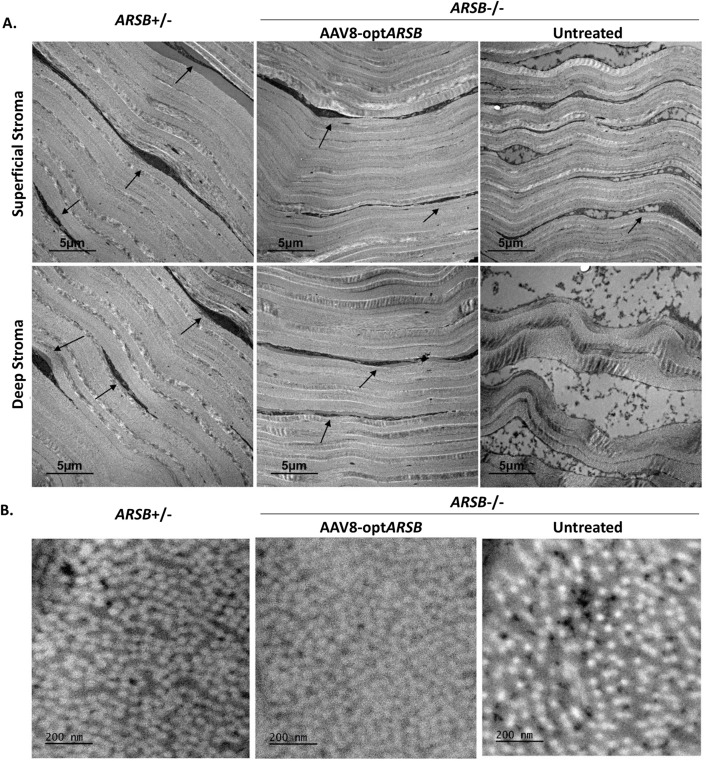
Electron microscopy images of corneas in MPS VI felines. Superficial and deep stroma on longitudinal sections (**A**) and cross sections (**B**) of MPS VI feline corneas were imaged with electron microscopy. Heterozygote (non-affected, *ARSB*^+/-^) and homozygote (affected MPS VI, *ARSB*^-/-^) felines with or without AAV8-opt*ARSB* intrastromal injection are imaged. Black arrows indicate corneal keratocytes, showing intracytoplasmic electron-lucent vacuoles in the corneal keratocytes in untreated homozygote feline cornea. Scale bars: 5 µm in (**A**) and 200 µm in (**B**).

Ultrastructural examination of the cornea via transmission electron microscopy revealed that the untreated superficial and posterior corneal stroma of a homozygous feline exhibited multiple intracytoplasmic electron-lucent vacuoles in the corneal keratocytes (Fig 5, keratocytes are indicated with black arrows), similar to the previously described granular inclusions observed in MPS VI affected felines [13]. Homozygous feline corneas treated with AAV8-opt*ARSB* showed far fewer of these granular inclusions along with less fibril disorganization of the posterior stroma compared to the untreated contralateral cornea (Fig 5).

**Fig 6:**
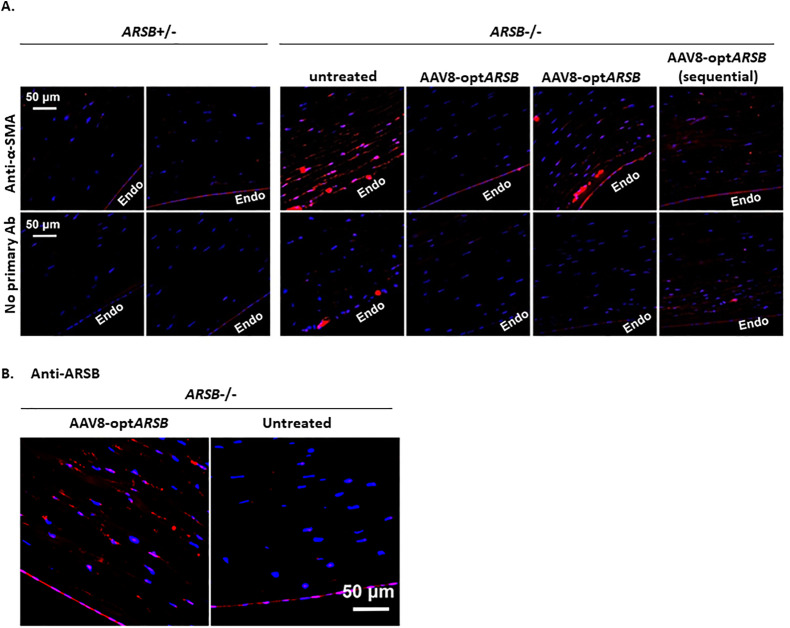
Immunofluorescence staining of corneas in MPS VI felines. Heterozygote (non-affected, *ARSB*^+/-^) and homozygote (affected MPS VI, *ARSB*^-/-^) feline corneas with or without AAV8-opt*ARSB* intrastromal injection were stained with anti-α-smooth muscle actin (α-SMA, a marker of fibrosis, (A)) or with anti-human arylsulfatase B (ARSB, (B)). Corneal stroma close to the endothelial side (Endo) was imaged. Scale bars: 50 µm.

Smooth muscle actin (SMA) staining, a marker of general fibrosis, showed heterozygous felines exhibited little to no fibrosis, whereas untreated homozygous felines demonstrated increased levels of SMA staining in the posterior stroma (Fig 6A). AAV8-opt*ARSB* treated homozygous corneas had less SMA staining than non-treated homozygous corneas (Fig 6A).

In addition, mild and diffuse GAG accumulation was noted throughout the corneal stroma with increased prevalence in the deep stroma of the central and peripheral cornea (S4 Fig). In all homozygote corneas treated with AAV8-opt*ARSB*, the central cornea was clear of GAG storage with mild diffuse corneal GAG storage in the peripheral cornea (S4 Fig, S3 Table).

### Human arylsulfatase is present in feline corneas upon postmortem analysis

To correlate the reversal of corneal opacity to the presence of the transgene product, immunofluorescence staining for human arylsulfatase B was performed. Both heterozygous and homozygous AAV8-opt*ARSB*-treated corneas were positive for diffuse arylsulfatase B staining in the feline corneal stroma compared to the untreated controls (Fig 6B, S5 Fig).

## Discussion

Adeno-associated virus based therapeutics for rare diseases face significant translational challenges, including high development costs associated with vector manufacturing, toxicology, and biodistribution studies. Streamlining this process using a well-characterized capsid, standardized injection parameters (dose, volume, and route), and a defined expression cassette is often constrained by the therapeutic payload and disease-specific anatomical and physiological considerations, including cell- or tissue-specific expression. However, the cornea represents a unique target tissue: it is compartmentalized, relatively immune-privileged, and anatomically relatively consistent across individuals. Moreover, the corneal stroma is composed primarily of quiescent keratocytes, and several natural AAV serotypes have demonstrated efficient transduction of this tissue. Long-term transgene expression following a single intrastromal injection under local anesthesia has been reported, supporting the feasibility of durable gene therapy in this compartment [7,18]. Therefore, a goal of the investigations herein was to capitalize on the extensive data from multiple species treating a similar lysosomal storage disease, aiming to accelerate the development of an AAV gene therapy pipeline for corneal diseases in general [10].

Previous studies have demonstrated the efficacy and safety of AAV-mediated gene addition to reverse MPS I-associated corneal clouding [7–9]. In alignment with this strategy, we employed the same AAV8 capsid, promoter, polyadenylation signal, vector dose, injection route, and volume to address corneal opacity in the most relevant MPS VI large animal model, MPS VI model felines [7–9]. To use MPS VI model felines, we performed 3 separate heterozygous breeding attempts, which resulted in only 2 homozygous affected offsprings. Even though this low sample size is a limitation of the current study, the corneal structure similarities with humans including the size and thickness along with directly translatable injection volume and vector doses [19] allow us to assess safety and efficacy of the gene therapy toward clinical trials applications. Intrastromal administration of AAV8-optARSB to post-symptomatic MPS VI felines resulted in resolution of corneal opacity within three weeks, with sustained clarity through the study endpoint and no adverse events. Importantly, overexpression of human *ARSB* in asymptomatic heterozygous MPS VI felines was well tolerated, indicating a favorable safety profile while highlighting the phenomenon of ocular immune privilege. These findings are consistent with prior observations in MPS I canines treated with AAV8-opt*IDUA*, where similar kinetics of corneal clearing were observed [7]. Together, these studies in two distinct MPS models and large animal species provide compelling evidence that intrastromal AAV gene therapy is a viable strategy for enzyme replacement in the cornea. Furthermore, the absence of inflammation or toxicity following AAV8-opt*ARSB* administration is consistent with findings from wild-type rabbit corneas treated with AAV8-opt*IDUA* at doses ten-fold higher than therapeutic levels, with no ocular toxicity observed over six months [9].

This study also demonstrates, for the first time in a large animal disease model, that contralateral sequential intrastromal AAV vector injections administered more than seven weeks apart are both safe and effective. This sequential dosing is clinically relevant, as MPS patients will likely require bilateral, sequential treatment to restore vision in both eyes. The reproducibility of corneal clearing kinetics in both eyes supports the robustness of this approach. Notably, the lack of immune interference suggests that corneal AAV vector administration may not preclude subsequent systemic or CNS-directed AAV-based therapies, which are under development and being evaluated in clinical trials for [20].

Clinical trials evaluating intravenous AAV8-*ARSB* for liver-directed therapy in MPS VI patients have demonstrated a favorable safety profile [20]. Our findings are consistent with these results, as human opt*ARSB* overexpression was well tolerated in vitro and in vivo. Plasmid-based opt*ARSB* expression did not impair cell viability or proliferation, and AAV8-mediated human opt*ARSB* expression in asymptomatic MPS VI felines was tolerated for the study duration. In symptomatic MPS VI felines, transient increases in CCT following a 50 μL intrastromal injection resolved within 24 hours, similar to asymptomatic subjects, suggesting that endothelial function is preserved despite disease pathology. Corneal clarity was restored within three weeks post-injection, with several notable observations.

First, storage disease clearing was restricted to the area of the cornea exposed to the injectate, consistent with arylsulfatase B’s primary localization to the lysosome and, to a lesser extent, the cellular membrane [21]. This observation necessitates direct delivery to affected regions. Consequently, peripheral corneal opacity and mild neovascularization persisted in the untreated areas. Future studies may require larger injection volumes or multiple injection sites to achieve a broader area of corneal coverage.

Second, a persistent observation in this study was that MPS VI feline corneas were thinner than those of heterozygous controls, despite the presence of GAG accumulation and increased collagen fibril spacing previously reported in this model [22]. This corneal thinning in MPS IV contrasts with MPS I canine models, where corneal thickening correlates with disease progression and GAG deposition. Notably, *ARSB*-deficient mice also exhibit thinner corneas compared to wild-type controls [23], suggesting a conserved phenotype across some species; however, this differs from MPS VI human patients, wherein CCT is increased with progressive disease [24]. The underlying mechanism remains unclear; however, the trend toward normalization of CCT and posterior stromal morphology following AAV8-opt*ARSB* therapy raises the possibility that these structural abnormalities are secondary to lysosomal storage pathology and may be reversible with longer-term correction. Alternatively, congenital anatomical differences may contribute to the observed phenotype. MPS VI felines exhibit craniofacial dysmorphia and reduced palpebral fissure length [14], which may be associated with inherently thinner corneas. A similar trend has been observed in human patients, where individuals with MPS VI exhibit corneal thicknesses comparable to those of unaffected controls [25]. In contrast, MPS I patients exhibit increased CCT, which correlates with opacity severity [26]. These interspecies and inter-disease differences underscore the importance of tailoring therapeutic strategies to the specific pathophysiology of each MPS subtype.

The collective data herein support the use of AAV8-opt*ARSB* corneal gene addition to alleviate vision loss due to MPS VI corneal clouding. Importantly, sequential dosing of the contralateral corneal at a later disease stage and at a time point that would have allowed for the generation of a humoral immune response also reversed corneal storage disease with the same kinetics as the original treatment. The experiments described herein were designed based on other large animal corneal gene addition strategies that rely on identical AAV vector elements, except for the disease-specific encoded DNA. These repeated demonstrations in large animal models across different species and diseases support an expedited approval process and significantly lower cost for the development of corneal gene therapies using standardized vector elements.

## Materials and Methods

### Plasmid cloning and vector preparation

To construct the ARSB expression plasmid, a codon-optimized ARSB sequence was synthesized and purchased from Genscript. The fragment was then PCR-amplified with the addition of NcoI and BamHI and cloned into the NcoI and BamHI sites of the pTR-EF1α-GFP vector. The construct was verified by restriction digestion and sequencing. The University of North Carolina Viral Vector Core prepared the AAV8-optARSB viral constructs. An alkaline gel was performed to confirm the integrity of the vector genome.

### Arylsulfatase B Activity Assay

ARSB activity assays were performed according to previously published methods [15]. Briefly, HEK293 cells were transfected with plasmids overexpressing *ARSB* or *GFP* with polyethyleneimine (PEI). Cell pellets were harvested, washed with phosphate-buffered saline (PBS), and then lysed in an appropriate volume of 0.15 M sodium acetate buffer. At least 5–10 freeze/thaw cycles were performed on the lysate alternating between a 37°C water bath and an ethanol/dry ice bath. Debris was removed by centrifugation at 12,000 rpm for 5 minutes. The total protein concentration was measured for each sample using the Bio-Rad Protein Concentration Assay (Bio-Rad, Hercules, CA). For each sample, 40 µL of lysate were added to 80 µL of substrate solution (0.15M sodium acetate buffer containing a final concentration of 3 mM lead acetate, 5 mM 4-methylumbeliferyl sulfate potassium salt [Sigma, St. Louis, MO], and 0.3 mM silver nitrate) in a clear bottom 96 well plate. Samples were incubated with substrate over a range of time periods, from 3 to 24 hours. Using 200 µL of stop buffer (0.5 M sodium bicarbonate, 0.5 M sodium carbonate, pH 10.32), the reaction was quenched, and fluorescence was measured on a Victor3 1420 Multilabel counter at an excitation of 365 nm, using an emission filter of 445 nm (PerkinElmer, Akron, OH). Results are expressed as nmol of 4-methylumbeliferyl sulfate (MUS)/µg of total protein/hour and are an average of two independent experiments with at least three replicates per experiment.

### Cytotoxicity assay

An alamarBlue viability assay assessed cytotoxicity. HEK293 cells were seeded at 150,000 cells per well into a 24-well plate. 24 hours later, 1.5 µg of CMV-*GFP* or EF1α-opt*ARSB* plasmids were transfected using PEI. Etoposide (20 µM) was used as a positive control for cytotoxicity. 72 hours after transfection, 10X alamarBlue solution (0.15 mg/mL resazurin sodium salt in PBS) was added to each well. After 3 hours of incubation, fluorescence (excitation = 540 nm; emission = 585 nm) of each well was measured on the BioTek Cytation 5 Imaging Reader system (Agilent, Santa Clara, CA, USA).

### Intra-corneal injection and clinical assessments in MPS VI Felines

The feline MPS VI colony was maintained at the University of Pennsylvania, School of Veterinary Medicine. Animals were raised under guidelines established by the National Institutes of Health (NIH) and the U.S. Department of Agriculture (USDA) for the care and use of animals in research. The genotypes of the animals were determined using a polymerase chain reaction (PCR)-based analysis. Two homozygous (male and female) and two heterozygous (male and female) felines for a null *ARSB* mutation (autosomal recessive, single point mutation [L476P]) [12] were transferred from the University of Pennsylvania to the North Carolina State University Laboratory Animal Resources at 75 days of age.

Their care and treatment were approved and monitored by the university institutional animal use and care committee (IACUC; approval #19-078-8). Use of the felines in these studies adhered to the Association for Research in Vision and Ophthalmology guidelines for the use of animals in ophthalmic research. All 4 subjects were monitored twice daily for animal health and behavior checkups and had complete ocular and physical examinations weekly until euthanasia at 242 days of age (90 days after initial injection), as defined by the protocol’s endpoint and required due to the progression of neurologic and orthopedic disease in the homozygous felines. Humane endpoint criteria to perform euthanasia before the protocol’s endpoint was based on the IACUC guideline, which was if that animal had ocular or systemic discomfort and it could not be resolved with treatment by 24 hours, then the animal would be euthanized. This did not occur with the cats of this report and none found dead in this study. Euthanasia was performed via intravenous injection of euthanasia solution (pentobarbital sodium and phenytoin sodium, EUTHASOL® Virbac AH, Inc.), per American Veterinary Medical Association guidelines. All husbandry and research staff were trained on the care, handling, and monitoring of felines. Staff had training through licensing as a registered veterinary technician, Laboratory Animal Technician, or doctor of veterinary medicine. Further training of the staff was conducted by the university laboratory animal veterinarian and the principal investigator, a veterinary ophthalmologist.

For the corneal injections, the felines were tranquilized with Dexmedetomidine (0.2 ml/5 kg) and ketamine (0.2 ml/5 kg). The eyes were aseptically prepped for surgery using topical 5% betadine, sterile eye wash, and topical 0.5% proparacaine. Felines were positioned under an operating microscope and were injected into the central corneal stroma using a 31-gauge needle introduced tangentially into the cornea to deliver 1e^9^ vg (50 µL) of AAV8-opt*ARSB* or saline to the axial, central corneal stroma. The injections and the subsequent clinical examinations were performed by a veterinary ophthalmologist (B.G.), who was masked to the identity of the test article administered. Following the injection, the felines received a topical ocular drop of moxifloxacin ophthalmic solution (0.5% Vigamox, Alcon) and buprenorphine (0.2 ml/5 kg SQ) for pain control and then were allowed to recover from the tranquilizer. Injection tolerability and corneal pathology were evaluated daily for the first 7 days after injection and then weekly. Clinical examinations included slit lamp biomicroscopy (KOWA SL-17, KOWA USA, Torrence, CA), indirect ophthalmoscopy (Vantage, Keeler USA, Malvern, USA) and tonometry. Clinical corneal score, a semi-quantitative assessment of corneal pathology, consisted of the sum of scores ranging from 0–4 (normal to most severe) of the following parameters: corneal opacity extent and areas involved in the opacity and corneal vascularization score ranging from 0–2 (no vascularization to severe).

### In-life corneal thickness and morphology assessments in MPS VI Felines

CCT was measured at each ophthalmic examination time point using an ultrasonic pachymeter (Pachpen, Accutome, Malvern, PA) following topical application of an anesthetic (Proparacaine HCL 0.5%, Alcaine, Alcon, Fort Worth, TX).

Spectral domain OCT (SD-OCT) was performed to image the cornea of each eye (Envisu R-class SD-OCT; Bioptigen, Inc., Morrisville, NC). Imaging was performed using the hand-held probe of the SD-OCT device fitted with a noncontact 12-mm telecentric lens for image acquisition. SD-OCT was set to 1000 A scans per B scan, and 100 B scans in total for each eye to generate a radial volume of 8 mm in diameter. B-scans and *en face* reconstructed images were reviewed. Corneas of all felines were imaged with SD-OCT from 99–204 days of age (before the second AAV8-opt*ARSB* injection in the sequential dosing cohort).

Felines were examined using *in vivo* confocal microscopy (Heidelberg Retina Tomograph 3 with Rostock Corneal Module, Heidelberg Engineering, GmbH, Dossenheim, Germany) of the cornea on day 242 of age. Corneas were imaged tangentially from the epithelium to the endothelium. Corneal endothelial counts were performed on the endothelial images of the central cornea using the automated feature of the Heidelberg system.

### Histologic evaluations and Immunofluorescence staining

Feline corneas were fixed, embedded in paraffin, sectioned at a thickness of 5 μm, and stained with hematoxylin, eosin, and alcian blue to assess GAG accumulation, or Masson’s trichrome to detect fibrosis, before examination by light microscopy. Histology scores were evaluated using the hematoxylin and eosin, alcian blue, and Masson’s trichrome stained sections. For immunofluorescence staining, paraffin-embedded tissues were deparaffinized and rehydrated before antigen retrieval procedures. Non-specific staining was blocked by using PBS containing 10% of normal goat serum, 0.025% Triton X-100, plus 1% BSA before overnight incubation with anti-ARSB antibody (dilutions: 1:100, Novus) or anti-alpha-Smooth Muscle Actin antibody (Novusbio, NBP1–30894). After incubation with fluorescence-conjugated secondary antibody (Alexa Fluor® 594, dilutions: 1:1000, Abcam), nuclei were counterstained with ProLong™ Diamond Antifade Mountant with DAPI (Invitrogen) and observed using an Olympus Fluorescence Microscope (Olympus, Tokyo, Japan).

### Transmission electron microscope

Treated and untreated corneas from MPS VI felines, and one control from a normal feline, were imaged with transmission electron microscopy (TEM). To obtain electron micrographs, tissues initially fixed in 4% paraformaldehyde, processed, and embedded in paraffin blocks were dewaxed and processed according to the protocol below. 2 mm x 2 mm paraffin blocks were cut and dewaxed in a xylene bath at 60°C and then rehydrated in a graded ethanol series. They were washed in 0.1 M sodium cacodylate, followed by post-fixation with 1% OsO_4_ at room temperature for 1 hour. Thick sections were cut at ~500 μm and stained with toluidine blue to check the tissue orientation and integrity. Samples were trimmed to an area small enough for a TEM grid and cut at ~90 nm thickness. The tissues were then washed with diH_2_O and dehydrated in a series of ethanol concentrations, from 50% to 100%. They were then stained with uranyl acetate and lead citrate for 30 min at room temperature. Dehydration was done through a graded ethanol series, followed by 100% propylene oxide, 50:50 Propylene Oxide/EPON, 25:75 Propylene Oxide/EPON, and 100% EPON. Sections were examined and photographed using a Tecnai 12 transmission electron microscope equipped with a Gatan Rio16 camera. The morphology of the anterior, middle, and posterior portions of the cornea was examined and photographed at an initial magnification of 1650x. The ultrastructure and arrangement of the collagen fibrils in each of the corneal regions were evaluated and photographed at a magnification of 44000X.

### Statistical analysis

Statistical analysis was performed using computerized statistical software (GraphPad Prism, version 10.2.3, GraphPad Software Inc., La Jolla, CA). Statistical significance for ARSB activity assay was assessed using a two-sample Student’s t-test with equal variance, as determined by an F test. Statistical significance for the cytotoxicity assay was evaluated using Tukey’s multiple comparisons test. Values of p < 0.05 were considered statistically significant.

## Supporting information

S1 Table Summary of heterozygous breeding attempts. (DOCX)

S2 Table Endothelial cell counts by confocal microscopy. (DOCX)

S3 Table Histology scores. (DOCX)

S1 Fig Cytotoxicity assessment. (DOCX)

S2 Fig Corneal clearing after AAV8-optARSB in MPS VI felines. (DOCX)

S3 Fig Central corneal thickness and optical coherence tomography imaging of the corneas. (DOCX)

S4 Fig Alcian blue staining of corneas in MPS VI felines. (DOCX)

S5 Fig Immunofluorescence staining of corneas in *ARSB* + /- felines. (DOCX)

## References

[1] Ashworth JL, Biswas S, Wraith E, Lloyd IC. The ocular features of the mucopolysaccharidoses. Eye (Lond). 2006;20(5):553–63. doi: 10.1038/sj.eye.6701921 15905869

[2] Taylor M, Khan S, Stapleton M, Wang J, Chen J, Wynn R, et al. Hematopoietic Stem Cell Transplantation for Mucopolysaccharidoses: Past, Present, and Future. Biol Blood Marrow Transplant. 2019;25(7):e226–46. doi: 10.1016/j.bbmt.2019.02.012 30772512 PMC6615945

[3] Koseoglu ST, Harmatz P, Turbeville S, Nicely H. Reversed papilledema in an MPS VI patient with galsulfase (Naglazyme) therapy. Int Ophthalmol. 2009;29(4):267–9. doi: 10.1007/s10792-008-9213-7 18418554 PMC2714452

[4] Turbeville S, Nicely H, Rizzo JD, Pedersen TL, Orchard PJ, Horwitz ME, et al. Clinical outcomes following hematopoietic stem cell transplantation for the treatment of mucopolysaccharidosis VI. Mol Genet Metab. 2011;102(2):111–5. doi: 10.1016/j.ymgme.2010.09.010 20980181 PMC3367500

[5] McGrath O, Au L, Ashworth J. Management of Corneal Clouding in Patients with Mucopolysaccharidosis. J Clin Med. 2021;10(15):3263. doi: 10.3390/jcm10153263 34362047 PMC8348690

[6] Entchev E, Antonelli S, Mauro V, Cimbolini N, Jantzen I, Roussey A, et al. MPS VI associated ocular phenotypes in an MPS VI murine model and the therapeutic effects of odiparcil treatment. Mol Genet Metab. 2022;135(2):143–53. doi: 10.1016/j.ymgme.2021.07.008 34417096

[7] Miyadera K, et al. Intrastromal gene therapy prevents and reverses advanced corneal clouding in a canine model of mucopolysaccharidosis I. Mol Ther. 2020;28(6):1455–63. 32330426 10.1016/j.ymthe.2020.04.004 PMC7264440

[8] Vance M, et al. AAV Gene Therapy for MPS1-associated Corneal Blindness. Sci Rep. 2016;6:22131. 26899286 10.1038/srep22131 PMC4761992

[9] Song L, Bower JJ, Llanga T, Salmon JH, Hirsch ML, Gilger BC. Ocular Tolerability and Immune Response to Corneal Intrastromal AAV-IDUA Gene Therapy in New Zealand White Rabbits. Mol Ther Methods Clin Dev. 2020;18:24–32. doi: 10.1016/j.omtm.2020.05.014 32542182 PMC7284066

[10] Bastola P, et al. Adeno-Associated Virus Mediated Gene Therapy for Corneal Diseases. Pharmaceutics. 2020;12(8). 10.3390/pharmaceutics12080767 PMC7464341 32823625

[11] Gilger BC, et al. A chimeric anti-vascularization immunomodulator prevents high-risk corneal transplantation rejection via ex vivo gene therapy. Mol Ther. 2024;32(11):4006–20. 39245940 10.1016/j.ymthe.2024.09.007 PMC11573577

[12] Yogalingam G, Litjens T, Bielicki J, Crawley AC, Muller V, Anson DS, et al. Feline mucopolysaccharidosis type VI. Characterization of recombinant N-acetylgalactosamine 4-sulfatase and identification of a mutation causing the disease. J Biol Chem. 1996;271(44):27259–65. doi: 10.1074/jbc.271.44.27259 8910299

[13] Haskins ME, Aguirre GD, Jezyk PF, Patterson DF. The pathology of the feline model of mucopolysaccharidosis VI. Am J Pathol. 1980;101(3):657–74. 6778219 PMC1903664

[14] Aguirre G, Stramm L, Haskins M. Feline mucopolysaccharidosis VI: General ocular and pigment epithelial pathology. Invest Ophthalmol Vis Sci. 1983;24(8):991–1007. 6409835

[15] Chang PL, Rosa NE, Davidson RG. Differential assay of arylsulfatase A and B activities: a sensitive method for cultured human cells. Anal Biochem. 1981;117(2):382–9. doi: 10.1016/0003-2697(81)90795-8 6119929

[16] Moodie KL, Hashizume N, Houston DL, Hoopes PJ, Demidenko E, Trembly BS, et al. Postnatal development of corneal curvature and thickness in the cat. Vet Ophthalmol. 2001;4(4):267–72. doi: 10.1046/j.1463-5216.2001.00198.x 11906662

[17] Alzubaidi R, Sharif MS, Qahwaji R, Ipson S, Brahma A. In vivo confocal microscopic corneal images in health and disease with an emphasis on extracting features and visual signatures for corneal diseases: a review study. Br J Ophthalmol. 2016;100(1):41–55. doi: 10.1136/bjophthalmol-2015-306934 26553917

[18] Hippert C, Ibanes S, Serratrice N, Court F, Malecaze F, Kremer EJ, et al. Corneal transduction by intra-stromal injection of AAV vectors in vivo in the mouse and ex vivo in human explants. PLoS One. 2012;7(4):e35318. doi: 10.1371/journal.pone.0035318 22523585 PMC3327666

[19] Gilger BC, Abarca E, Salmon JH. Selection of appropriate animal models in ocular research: ocular anatomy and physiology of common animal models. In: Gilger BC, editor. Ocular pharmacology and toxicology. Totowa, NJ: Humana Press; 2014. p. 7–32.

[20] Brunetti-Pierri N, Ferla R, Ginocchio VM, Rossi A, Fecarotta S, Romano R, et al. Liver-Directed Adeno-Associated Virus-Mediated Gene Therapy for Mucopolysaccharidosis Type VI. NEJM Evid. 2022;1(7):EVIDoa2200052. doi: 10.1056/EVIDoa2200052 38319253

[21] Prabhu SV, Bhattacharyya S, Guzman-Hartman G, Macias V, Kajdacsy-Balla A, Tobacman JK. Extra-lysosomal localization of arylsulfatase B in human colonic epithelium. J Histochem Cytochem. 2011;59(3):328–35. doi: 10.1369/0022155410395511 21378286 PMC3201152

[22] Alroy J, Haskins M, Birk DE. Altered corneal stromal matrix organization is associated with mucopolysaccharidosis I, III and VI. Exp Eye Res. 1999;68(5):523–30. doi: 10.1006/exer.1998.0622 10328965

[23] Strauch OF, Stypmann J, Reinheckel T, Martinez E, Haverkamp W, Peters C. Cardiac and ocular pathologies in a mouse model of mucopolysaccharidosis type VI. Pediatr Res. 2003;54(5):701–8. doi: 10.1203/01.PDR.0000084085.65972.3F 12904606

[24] Ahmed TY, Turnbull AMJ, Attridge NF, Biswas S, Lloyd IC, Au L, et al. Anterior segment OCT imaging in mucopolysaccharidoses type I, II, and VI. Eye (Lond). 2014;28(3):327–36. doi: 10.1038/eye.2013.281 24384963 PMC3965814

[25] Kottler U, Demir D, Schmidtmann I, Beck M, Pitz S. Central corneal thickness in mucopolysaccharidosis II and VI. Cornea. 2010;29(3):260–2. doi: 10.1097/ICO.0b013e3181b55cc1 20098308

[26] Connell P, McCreery K, Doyle A, Darcy F, O’Meara A, Brosnahan D. Central corneal thickness and its relationship to intraocular pressure in mucopolysaccararidoses-1 following bone marrow transplantation. J AAPOS. 2008;12(1):7–10. doi: 10.1016/j.jaapos.2007.04.003 17588792

