# Supplementary material for: Adeno-associated vector corneal gene therapy reverses corneal clouding in a feline model of mucopolysaccharidosis VI
Source: PLoS One. 2025 Dec 5;20(12):e0338370. doi: 10.1371/journal.pone.0338370 (PMC12680226; doi:10.1371/journal.pone.0338370)
Supplement: S1 Table — (DOCX) [file pone.0338370.s001.docx]

**Supporting Information**

**S1 Table. Summary of heterozygous breeding attempts.**

| Breeding  attempt | Normal | Heterozygote (non-affected) | Homozygote (affected) | Found Dead |
| --- | --- | --- | --- | --- |
| First | 1 | 1 | 1 | 0 |
| Second | 0 | 1 | 0 | 2 |
| Third | 3 | 2 | 1 | 0 |

Three separate heterozygous breeding attempts resulted in 10 offspring, 2 of which were homozygous for the L476P mutation and demonstrated an MPS VI phenotype.
