# Supplementary material for: Adeno-associated vector corneal gene therapy reverses corneal clouding in a feline model of mucopolysaccharidosis VI
Source: PLoS One. 2025 Dec 5;20(12):e0338370. doi: 10.1371/journal.pone.0338370 (PMC12680226; doi:10.1371/journal.pone.0338370)
Supplement: S2 Table — (DOCX) [file pone.0338370.s002.docx]

**Supporting Information**

**S2 Table. Endothelial cell counts by confocal microscopy.**

| Genotype (phenotype) | Subject number | Untreated or Saline | AAV8-optARSB |
| --- | --- | --- | --- |
| Homozygote (affected) | Subject #1 | 2215 | 2493 |
|  | Subject #2 |  | 3179 |
|  |  |  | *3015 |
| Heterozygote (non-affected) | Subject #3 | 2271 | 2831 |
|  | Subject #4 | 2329 |  |

Endothelial cell counts were performed on day 242 of age, which is 90 days after the dosing, and is 36 days after the sequentially dosed eye.

*Sequentially dosed eye. This eye was dosed with saline at day 152 of age and with AAV8-opt*ARSB* at day 206 of age.
