# Supplementary material for: Adeno-associated vector corneal gene therapy reverses corneal clouding in a feline model of mucopolysaccharidosis VI
Source: PLoS One. 2025 Dec 5;20(12):e0338370. doi: 10.1371/journal.pone.0338370 (PMC12680226; doi:10.1371/journal.pone.0338370)
Supplement: S3 Table — (DOCX) [file pone.0338370.s003.docx]

**Supporting Information**

**S3 Table. Histology scores**

|  |  |  | **Central cornea** | | | | **Peripheral cornea** | | | |
| --- | --- | --- | --- | --- | --- | --- | --- | --- | --- | --- |
| **Subject number**  **/eye** | **Genotype (Phenotype)** | **Treatment**  **group** | **Cell infiltrate** | **Vascular-ization** | **Storage disease (alcian blue)** | **Fibrosis**  **/scaring**  **(Masson’s Trichrome)** | **Cell infiltrate** | **Vascular-ization** | **Storage disease (alcian blue)** | **Fibrosis**  **/scaring**  **(Masson’s Trichrome)** |
| Subject #1/OS | *ARSB*^-/-^ (affected) | No treatment | 0 | 0 | 2 | 0 | 0 | 1 | 2 | 0 |
| Subject #1/OD | *ARSB*^-/-^ (affected) | AAV8-opt*ARSB* | 0 | 0 | 0 | 0 | 0 | 0 | 2  (nasal only) | 0 |
| Subject #2/OS | *ARSB*^-/-^ (affected) | AAV8-opt*ARSB* | 0 | 0 | 0 | 0 | 0 | 0 | 1 | 0 |
| Subject #2/OD | *ARSB*^-/-^ (affected) | AAV8-opt*ARSB* (sequential) | 0 | 0 | 0 | 0 | 0 | 1 | 2 | 0 |
| Subject #3/OS | *ARSB*^+/-^ (non-affected) | Saline | 0 | 0 | 0 | 0 | 0 | 0 | 0 | 0 |
| Subject #3/OD | *ARSB*^+/-^ (non-affected) | AAV8-opt*ARSB* | 0 | 0 | 0 | 0 | 0 | 0 | 0 | 0 |
| Subject #4/OS | *ARSB*^+/-^ (non-affected) | No treatment | 0 | 0 | 0 | 0 | 0 | 0 | 0 | 0 |
| Subject #4/OD | *ARSB*^+/-^ (non-affected) | AAV8-opt*ARSB* | 0 | 0 | 0 | 0 | 0 | 0 | 0 | 0 |

Histology scores of the corneas were evaluated using the hematoxylin and eosin, alcian blue, and Masson’s trichrome stained sections. Scores were evaluated for cell infiltrate (with hemotoxylin and eosin staining), vascularization (with hematoxylin and eosin staining), glycosaminoglycans accumulation (storage disease, with alcian blue staining) and fibrosis/scaring (with Masson’s trichrome staining) with scores as follows: 0 = normal/none; 1 = Mild focal; 2 = Mild diffuse or Moderate focal; 3 = Moderate diffuse or Severe focal; 4 = Severe diffuse. OS: left eye, OD: right eye.
