# Supplementary material for: Adeno-associated vector corneal gene therapy reverses corneal clouding in a feline model of mucopolysaccharidosis VI
Source: PLoS One. 2025 Dec 5;20(12):e0338370. doi: 10.1371/journal.pone.0338370 (PMC12680226; doi:10.1371/journal.pone.0338370)
Supplement: S1 Fig — (DOCX) [file pone.0338370.s004.docx]

**Supporting Information**

**
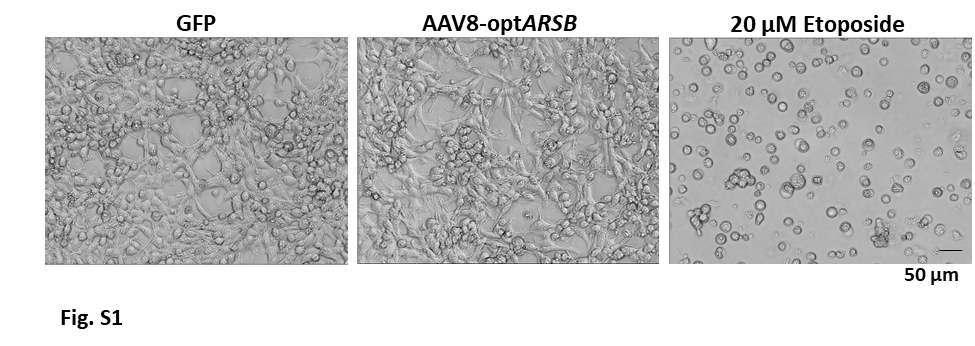
S1 Figure. Cytotoxicity assessment.** HEK293 cells were transfected with the opt*ARSB* plasmid or GFP plasmid (control). A concentration of 20 µM etoposide was used as a cytotoxicity positive control. Cells were imaged 72 hours after transfection or the addition of etoposide. Scale bar: 50 µm.
