# Supplementary material for: Adeno-associated vector corneal gene therapy reverses corneal clouding in a feline model of mucopolysaccharidosis VI
Source: PLoS One. 2025 Dec 5;20(12):e0338370. doi: 10.1371/journal.pone.0338370 (PMC12680226; doi:10.1371/journal.pone.0338370)
Supplement: S2 Fig — (DOCX) [file pone.0338370.s005.docx]

**Supporting Information**


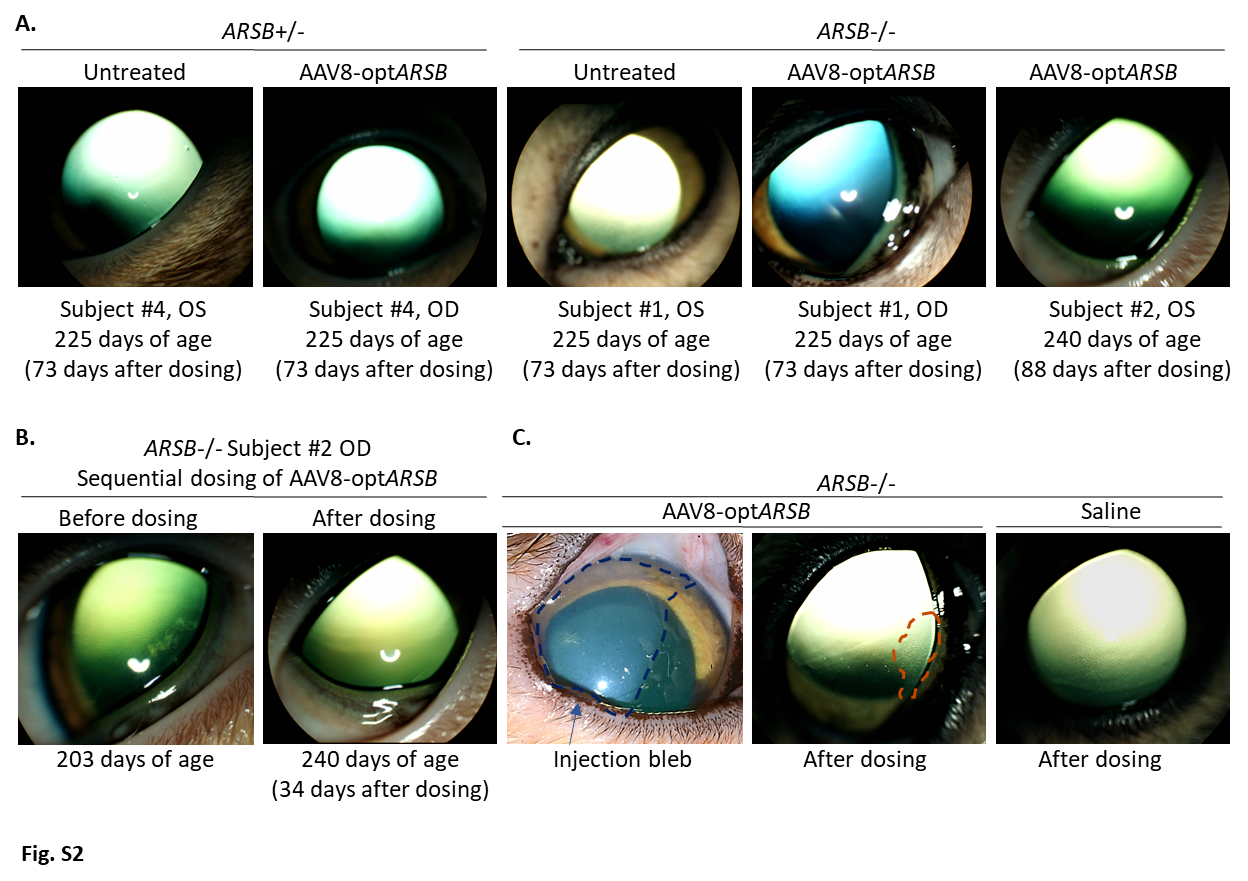


**Figure S2.** **Corneal clearing after AAV8-optARSB in MPS VI felines.** (**A**) Corneas from felines homozygous for the L476P mutation in the *ARSB* gene (*ARSB*^-/-^, affected MPS VI felines) and heterozygous feline cornea (*ARSB*^+/-^, not affected control felines) with or without AAV8-opt*ARSB* (1e^9^ vg) intrastromal injection were imaged. (**B**) Homozygote cornea (Subject #2, OD) was dosed with AAV8-opt*ARSB* intrastromal injection following preceding AAV8-opt*ARSB* injection to the contralateral eye (sequential dosing), and imaged before and after the sequential dosing. OD: right eye, OS: left eye. (**C**) Homozygote corneas dosed with AAV8-opt*ARSB* (left and middle photos) or saline (right photo) intrastromal injection were imaged. The clear area of the AAV8-opt*ARSB* treated cornea (remnant cloudy corneal area was indicated with red dotted line in the middle photo) showed co-localization with the area where injection bleb was observed immediately after the injection (indicated with blue dotted line and blue arrow in the left photo). Saline injected cornea showed corneal cloudiness (right photo).
