# Supplementary material for: Adeno-associated vector corneal gene therapy reverses corneal clouding in a feline model of mucopolysaccharidosis VI
Source: PLoS One. 2025 Dec 5;20(12):e0338370. doi: 10.1371/journal.pone.0338370 (PMC12680226; doi:10.1371/journal.pone.0338370)
Supplement: S3 Fig — (DOCX) [file pone.0338370.s006.docx]

**Supporting Information**

**
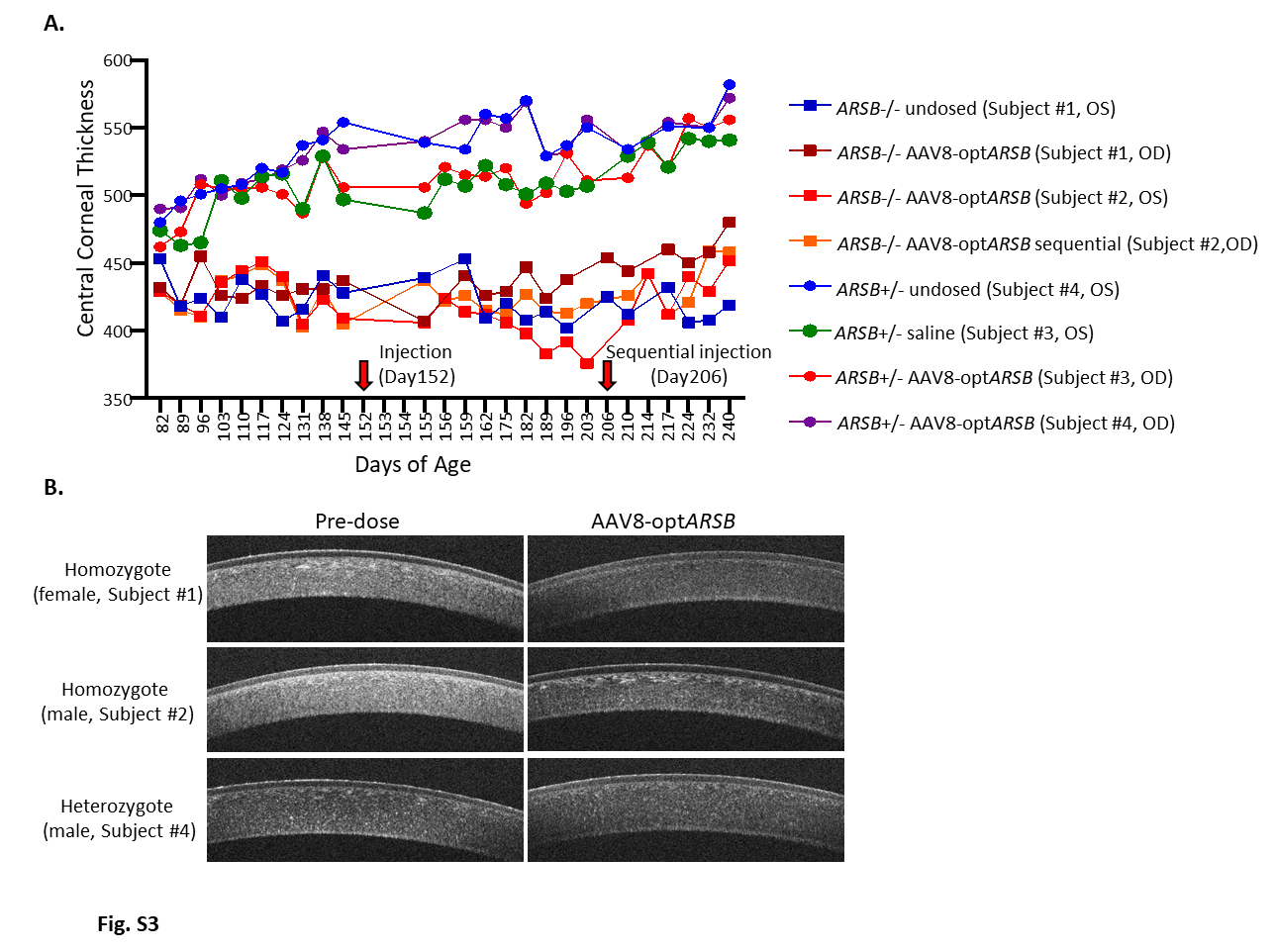
 Figure S3. Central corneal thickness and optical coherence tomography imaging of the corneas.** (**A**) Central corneal thickness was measured by ultrasonic pachymeter over the experiment period (from 82 to 240 days of age). The central corneal thickness for each eye is shown. Corneas were dosed with intrastromal AAV8-opt*ARSB* (1e^9^ vg) or saline injection on 152 days of age. One homozygous eye (Subject #2, OD) was dosed with AAV8-opt*ARSB* at 206 days of age following a preceding AAV8-opt*ARSB* injection on the contralateral eye (Subject #2, OS) on 152 days of age (sequential dosing). OD: right eye, OS: left eye. (**B**) Representative optical coherence tomography images of the corneas. Images of corneas before injection (pre-dose) and 60 days after the AAV8-opt*ARSB* injection are shown.
