# Supplementary material for: Adeno-associated vector corneal gene therapy reverses corneal clouding in a feline model of mucopolysaccharidosis VI
Source: PLoS One. 2025 Dec 5;20(12):e0338370. doi: 10.1371/journal.pone.0338370 (PMC12680226; doi:10.1371/journal.pone.0338370)
Supplement: S4 Fig — (DOCX) [file pone.0338370.s007.docx]

**Supporting Information**

**
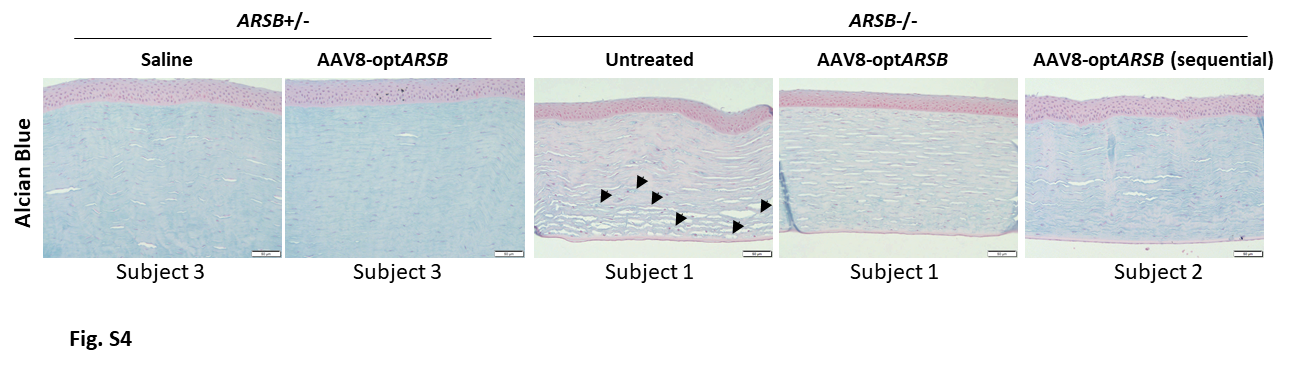
 Figure S4. Alcian blue staining of corneas in MPS VI felines.** Heterozygote (non-affected, *ARSB*^+/-^) and homozygote (affected MPS VI, *ARSB*^-/-^) feline corneas with or without AAV8-opt*ARSB* intrastromal injection were stained with alcian blue to assess glycosaminoglycans accumulation (black arrows). Scale bar: 50 µm.
