# Supplementary material for: Adeno-associated vector corneal gene therapy reverses corneal clouding in a feline model of mucopolysaccharidosis VI
Source: PLoS One. 2025 Dec 5;20(12):e0338370. doi: 10.1371/journal.pone.0338370 (PMC12680226; doi:10.1371/journal.pone.0338370)
Supplement: S5 Fig — (DOCX) [file pone.0338370.s008.docx]

**Supporting Information**


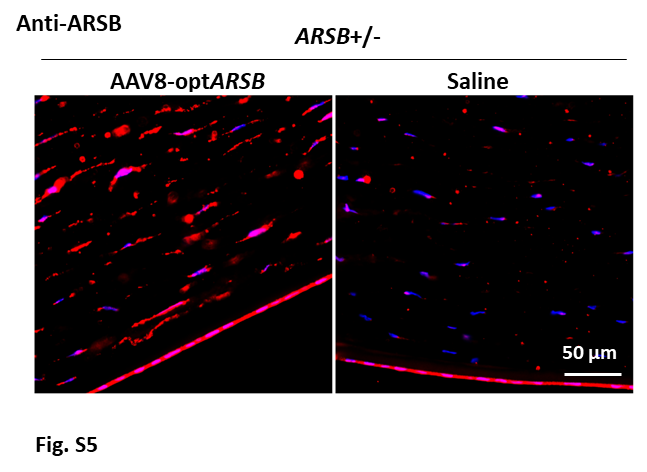


**Figure S5. Immunofluorescence staining of corneas in *ARSB*+/- felines.**

Heterozygote (non-affected, *ARSB*^+/-^) feline corneas with AAV8-opt*ARSB* or saline intrastromal injection were stained with anti-human arylsulfatase B (ARSB) antibody. Corneal stroma close to the endothelial side was imaged. Scale bar: 50 µm.
